# “Everyone Thought She Was Crazy”: A Report on a Novel Approach to Diagnosing a Case of Anti-NMDAR Encephalitis

**DOI:** 10.7759/cureus.27671

**Published:** 2022-08-04

**Authors:** Ricardo Martinez, Claudia Perez Acosta, Sarah Cormie

**Affiliations:** 1 Neurology, Larkin Community Hospital, Miami, USA; 2 Internal Medicine, Mount Sinai Medical Center, Miami, USA

**Keywords:** psychiatry patients, teratoma, nmdar antibodies, anti-n-methyl d-aspartate (nmda) receptor, multiple stages

## Abstract

We present a unique case of a 28-year-old female patient admitted to our hospital due to altered mental status, hallucinations, agitation, impaired memory, and impaired speech. The patient had a previous psychiatric admission to another hospital but she was discharged without a definitive diagnosis. During her admission, the majority of the medical staff was under the impression she was having a psychotic event. The patient was subsequently diagnosed with N-methyl-D-aspartate receptor (NMDAR) encephalitis. Our aim is to report the clinical approach to diagnose and manage a type of autoimmune encephalitis associated with ovarian teratomas, but most importantly to teach and make awareness in the medical community of how to recognize this type of encephalitis.

## Introduction

Anti-N-methyl D-aspartate (NMDA) receptor (anti-NMDAR) encephalitis is one of the most common causes of autoimmune encephalitis. The antibodies are immunoglobulin G (IgG) that target the GluN1 subunit of the N-methyl D-aspartate receptor (NMDAR). It is most commonly observed in young females and children but men can also be affected. This is a syndrome characterized by multiple stages. Usually, patients develop prodromic flu-like symptoms (fatigue, nausea, headache, and fever), two or three weeks prior. Signs and symptoms include psychiatric, seizures, memory deficits, decreased level of consciousness, dyskinesias, and autonomic instability. These patients are often confused with psychiatry patients and delay in the diagnosis is a frequent pattern. The majority of the patients require intensive care unit (ICU) care. This condition has a strong association with ovarian teratomas and thus is seen as more common in women. Approximately 40% of female patients over 18 years have uni- or bilateral ovarian teratomas compared to less than 9% of girls under 14 years of age [1,2]. As in our case, most patients recover, albeit slowly.

## Case presentation

We present a unique case of a 28-year-old female patient currently admitted to our hospital due to altered mental status, hallucinations, agitation, short-term memory loss, and effortful speech with trouble producing language giving us the impression of expressive aphasia. The local institutional review board does not require patient consent for case reports if the patient is sufficiently de-identified. Consent was not obtained from the participant in this case report.

**Figure 1:**
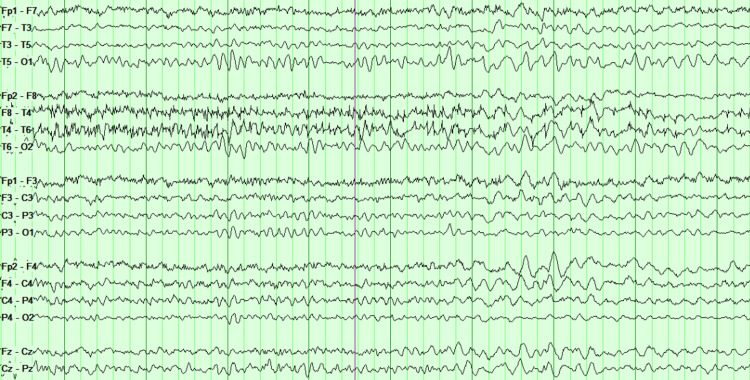
Bilateral cerebral dysfunction with generalized slowing (Theta/Delta).

Upon hospital admission, the initial workup was normal and she was transferred to the Behavioral Health Unit. She was found to have a history of multiple admissions the prior month to another local hospital due to severe headaches, neck stiffness, fever, and emesis. At that time, she underwent an extensive workup including a lumbar puncture which was negative, and she was subsequently discharged from the hospital. The neurology team was consulted, and the patient showed psychomotor slowing, although able to follow commands, perseveration, and orofacial dyskinesia were noted. Due to the patient’s sex, age, signs, symptoms, previous admission with no clear diagnosis, and no past medical history of psychiatric condition a lumbar puncture was performed. The brain imaging (CT scan and MRI) was negative. She was started on methylprednisolone 1000 mg daily for five days. EEG was performed and showed bilateral cerebral dysfunction but no epileptiform patterns (Figure 1).

**Figure 2:**
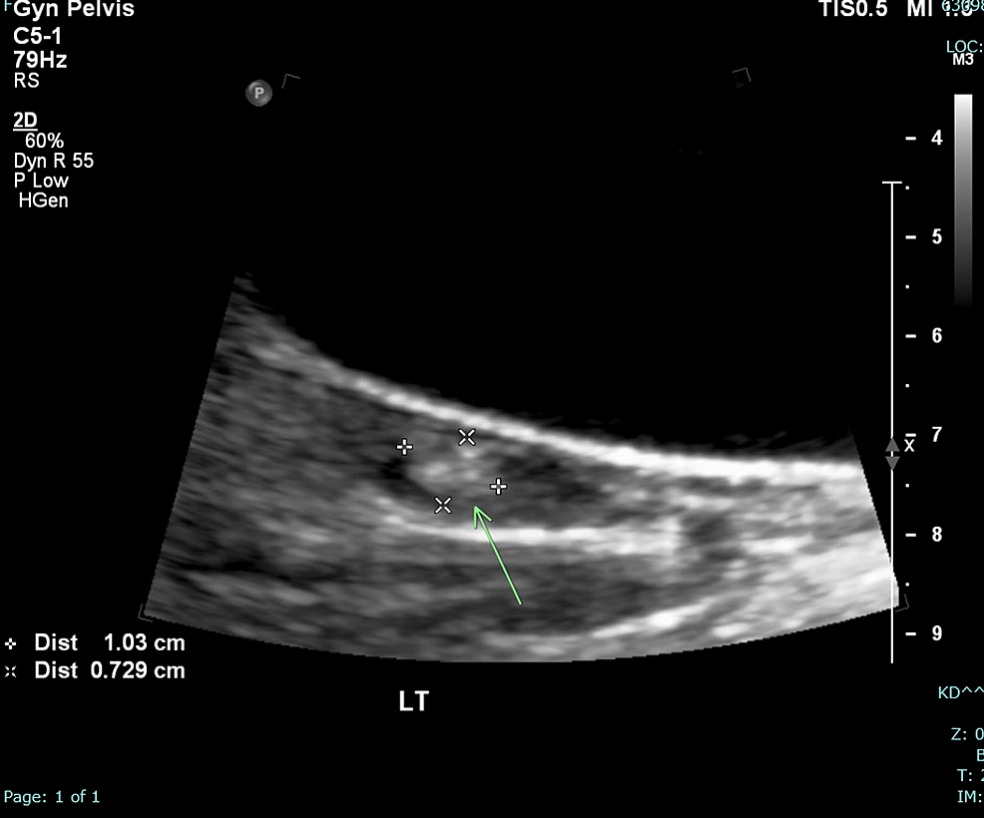
Nonobstetric pelvic ultrasound showing 1 cm hyperechoic left ovarian lesion.

At this point, the primary differential was autoimmune encephalitis therefore we continued intravenous (IV) steroids and initiated intravenous immunoglobulin (IVIG). She was transferred to the ICU for closer monitoring. In the ICU she started developing dystonic movements in all extremities. The patient’s heart rate ranged from 45-60 beats per minute. Laboratory workup returned with a positive antinuclear antibody (ANA) and positive NMDA receptor antibody in the CSF. The patient developed catatonia after 24 hours of admission to the ICU. A nonobstetric pelvic ultrasound showed a 1 cm hyperechoic left ovarian lesion (Figure 2).

**Figure 3:**
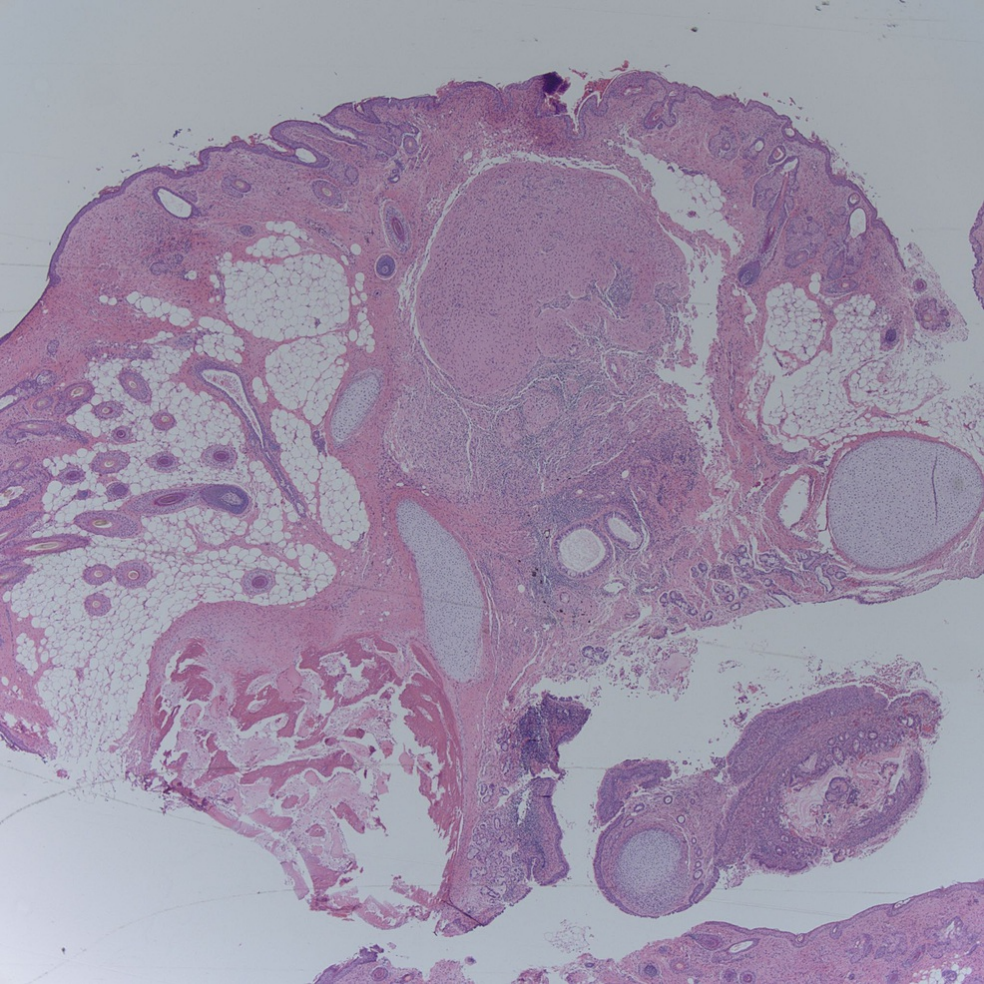
Mature teratoma histopathology image 1

**Figure 4:**
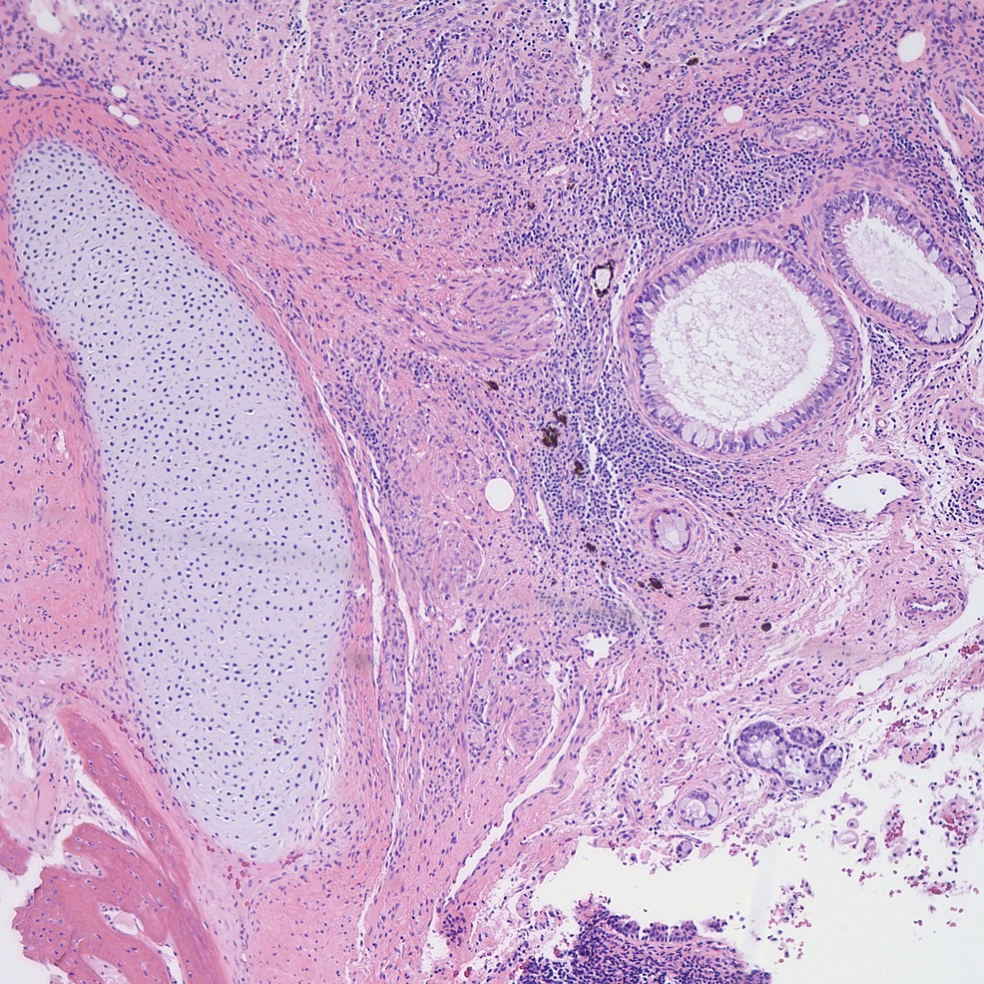
Mature teratoma histopathology image 2

**Figure 5:**
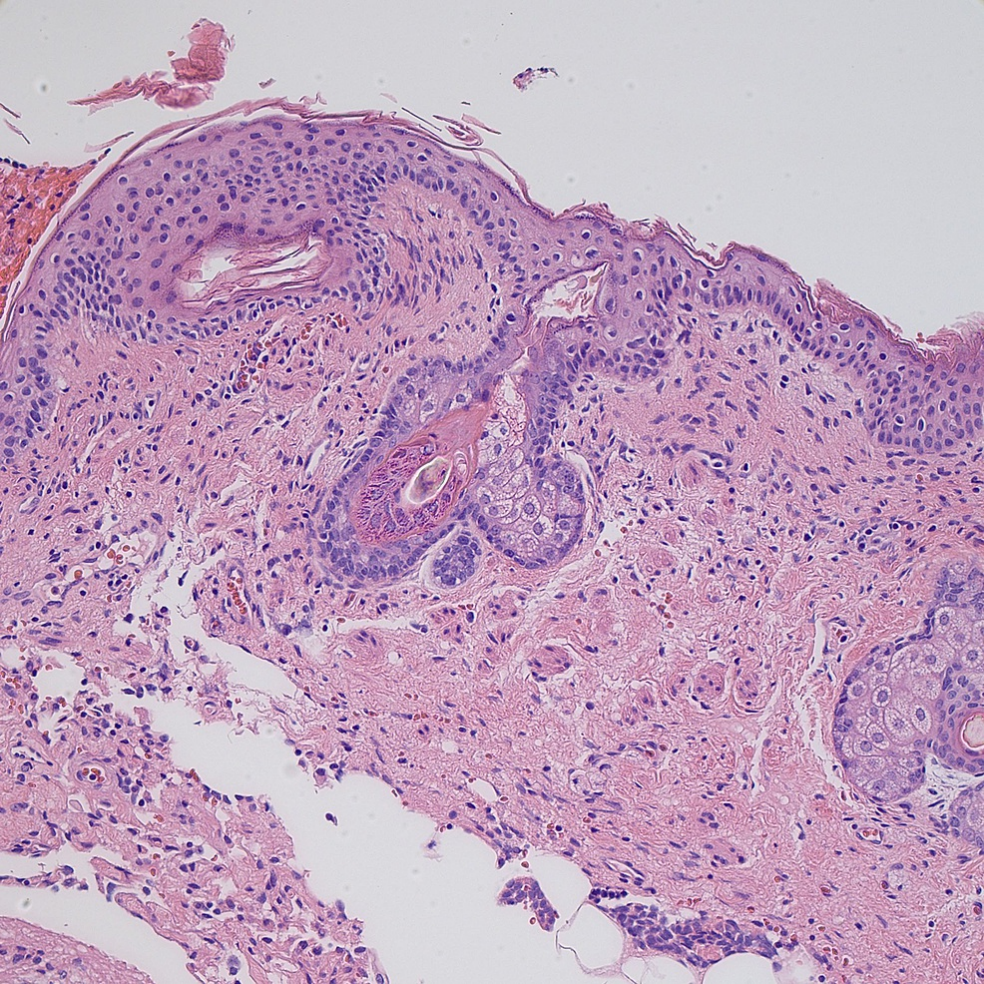
Mature teratoma histopathology image 3

**Figure 6:**
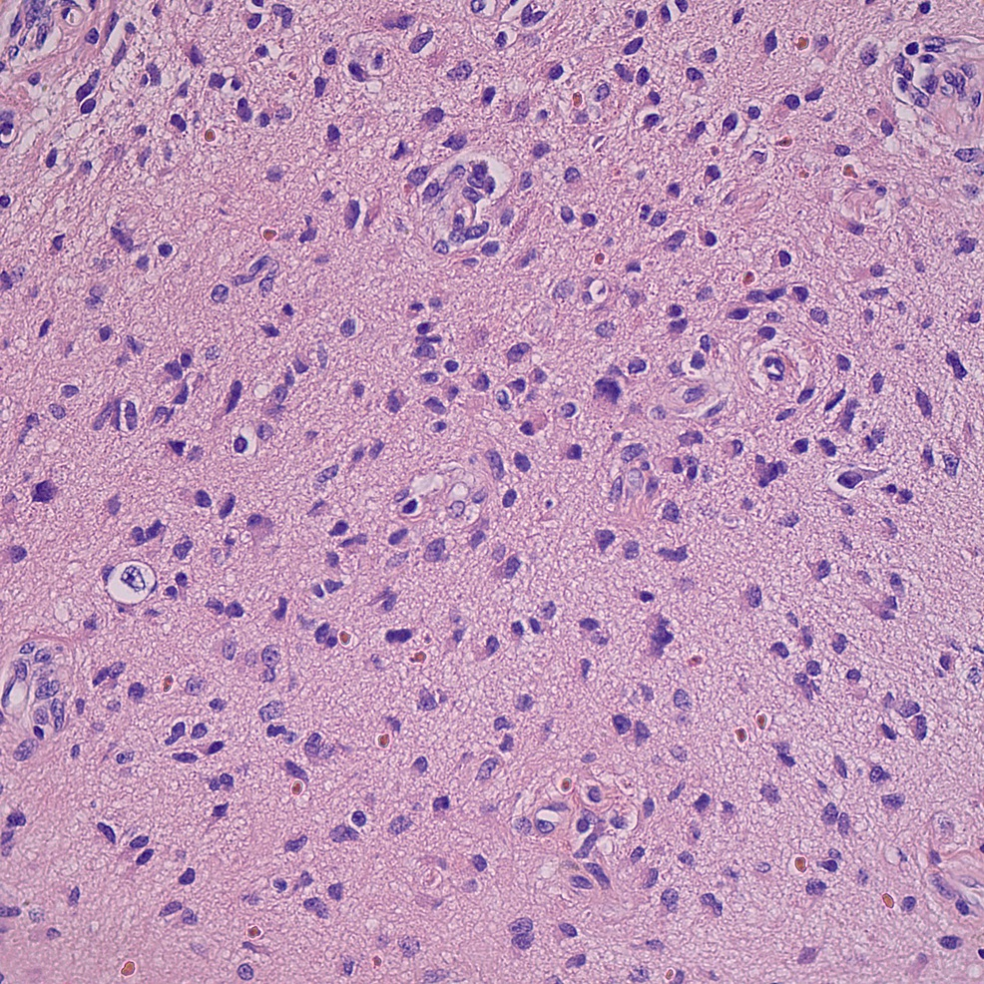
Mature teratoma histopathology image 4

She underwent a laparoscopic left salpingo-oophorectomy due to teratoma. Histopathology indicated a mature teratoma (Figure 3-6). The patient’s mental status has subsequently continued to improve [3].

## Discussion

One question that remains among many neurologists is whether an autoimmune encephalitis panel should become part of the initial workup for all patients with new-onset isolated psychiatric symptoms especially if they are otherwise healthy. In our experience, we think that while evaluating every patient with new onset of psychiatric symptoms should be aware of autoimmune encephalitis, although most patients will have subtle neurological or systemic symptoms. Physicians should have a low threshold to consider sending an antibody panel. We also think that the NMDAR antibodies should be tested in serum and CSF, with CSF being more specific. A common problem that many hospitals experience is the delay in the results of the antibody panel and the question that remains is if treatment should be initiated even without the results. After reviewing the available literature and based on our experience we concluded that if the pretest probability is high enough we shouldn’t wait for the results of the antibody panel to come back. Early treatment is very important in controlling the initial manifestations, and also improves the long-term outcome of the disease. We also think that we should strike hard early while treating these patients and we shouldn’t be too modest by only giving one regimen, so we concluded that starting a combination of therapies like steroids and intravenous immunoglobulin or steroids and plasma exchange depending on the experience of the center is the best approach. Plasma exchange might be quicker to see the results and intravenous immunoglobulin is a little bit easier to administer in patients with psychiatric manifestations [4].

After reviewing the literature we concluded that while waiting for the antibody results we also should have started this patient on intravenous acyclovir, as Herpes simplex encephalitis is another frequent etiology of autoimmune encephalitis [5].

Radiology of the brain is non-specific but may reveal abnormalities suggesting an autoimmune process, and brain MRI is the most sensitive modality. Brain MRI may reveal abnormal Fluid-Attenuated Inversion Recovery (FLAIR) or T2 (transverse relaxation time) hyperintensities in the medial temporal lobe, cerebral/cerebellar cortex, basal ganglia, and brainstem. Normal brain MRI is a common finding observed more frequently in female patients than in male patients [6]. 

Despite a growing body of literature, patients with anti-NMDAR encephalitis are frequently diagnosed with psychiatric conditions mainly during the initial stages. The incidence of psychiatric symptoms in this condition is 65-80%. Antibodies that are produced by ovarian teratoma cause dysfunction of the NMDAR and glutaminergic system. Studies have shown that hypofunction of the NMDAR may lead to secondary dopaminergic dysregulation. Although most patients with anti-NMDAR encephalitis have no prior psychiatric histories, there are cases that do have and the initial presentation may be mistaken for new-onset primary psychosis or pharmaceutical effects. It is very important to make an accurate diagnosis since this condition is treatable, but potentially fatal. The pattern of a young female patient, without a history of psychiatric events, prodromic flu-like symptoms, and multistage symptomatology is typical. Although there is no uniform standard for the diagnosis, the disease is confirmed by NMDAR antibodies in CSF and serum. Immediate initiation of immunosuppressive therapies and antibody depletion (tumor removal), is the cornerstone of the treatment [7].

## Conclusions

Based on our bibliographic review and professional experience, it is important to increase awareness in the medical community of possible organic and auto-immune etiology for psychiatric presentations and also be cautious that initial negative laboratory and imaging workups do not exclude organic causes. Anti-NMDA encephalitis is a condition that requires a high level of suspicion to make a diagnosis. In our experience, an interdisciplinary thought process must be implemented in order to consider this condition as a diagnosis. Timely treatment often leads to a good prognosis in patients.
